# Exercises in Clinical Reasoning: Applying High-Value Care When the Counts Don’t Add Up

**DOI:** 10.1007/s11606-026-10450-2

**Published:** 2026-04-20

**Authors:** Rohan Rao, Katya Lavine, Anand A. Patel, Christopher Moriates

**Affiliations:** 1 Department of Neurology, UCLA David Geffen School of Medicine, Los Angeles, CA USA; 2 Department of Medicine, UCLA David Geffen School of Medicine, Los Angeles, CA USA; 3 Section of Hematology/Oncology, Department of Medicine, University of Chicago, Chicago, IL USA; 4 Department of Medicine, VA Greater Los Angeles Healthcare System, Los Angeles, CA USA

In this series, a clinician extemporaneously discusses the diagnostic approach (regular text) to sequentially presented clinical information **(bold)**. Additional commentary on the diagnostic reasoning process (*italics*) is integrated throughout the discussion.

**A 78-year-old man with a history of severe lumbar stenosis, psoriatic arthritis, hemorrhoids, and metabolic dysfunction-associated fatty liver disease presented to the emergency department after being sent by his outpatient provider for worsening anemia. His hemoglobin (Hgb) was 7.2 g/dL on pre-operative work-up, decreased from a baseline of 10–11 g/dL 6 months prior. He reported increasing dyspnea on exertion that had been present for 1 year but had increased over the past week. He did not have any overt evidence of bleeding such as hematuria or melena. Review of systems was positive for chronic lower back pain due to severe lumbar stenosis.**

My general approach to anemia is to consider hypoproliferative etiologies (i.e., a hematologic malignancy such as leukemia or myelodysplastic syndrome (MDS) impacting bone marrow production, nutritional deficiency such as iron or B12, lack of erythropoietin production due to chronic kidney disease) versus hyperproliferative etiologies (i.e., causes such as gastrointestinal bleed, splenic sequestration, hemolysis that would stimulate a compensatory hematopoietic response from the bone marrow). Classifying the anemia by red cell size and factoring in the pace of disease is also helpful. An acute drop in hemoglobin is typically driven by loss while a subacute/chronic worsening of anemia is more consistent with decreased bone marrow production. The patient’s age raises my concern for malignancy as an etiology of anemia; this could be iron deficiency anemia from a colorectal cancer or a hematologic malignancy driving hypoproliferation. When considering the age of this patient, I would start by evaluating iron stores, ferritin, B12, and thyroid stimulating hormone (TSH).^1^ I would also get a complete blood count (CBC) with differential along with a peripheral blood smear to evaluate the presence of other cytopenias and any clues to a primary bone marrow pathology. Anemia can be multifactorial in etiology particularly in older adults, so factoring in relevant contributors for a specific patient is also important. Pertinent findings from the patient’s history include chronic liver disease, psoriatic arthritis, and internal hemorrhoids. Anemia, due to liver disease, can be multifactorial including gastrointestinal bleeding, splenic sequestration, and spur cell anemia. Psoriatic arthritis could be a contributor to anemia of inflammation, and internal hemorrhoids could be a source of occult blood loss. Next, etiologies that could tie together the patient’s worsening back pain and anemia, such as multiple myeloma, should be considered when interpreting the initial set of diagnostic labwork.

*The physician discussant used a broad framework to guide his approach and next steps. He considered the patient’s age, symptoms, and history to refine his diagnostic pathway, selecting relevant labs. High-value care aims to maximize patient-important outcomes while minimizing unnecessary costs and harms. Avoiding overtesting is an important principle of providing high-value care, reducing both physical and financial harms.*^2^ *Decisions on diagnostic tests should be based on whether they provide additional information beyond history, examination, and cost-effective tests.*^3^* For example, some routine anemia labs, like folate, are low-value due to poor test performance and the rarity of deficiency in the post-fortification era.*^4^

**Social history was notable for 20-pack-year smoking history with cessation 1 year prior. Patient denied alcohol use. Surgical history was pertinent for left nephrectomy after donation to his sister in 2001.**

**His medications were oral diclofenac, lidocaine patches, and acetaminophen/hydrocodone for back pain; methotrexate, prednisone 4 mg daily, and upadacitinib for psoriatic arthritis; tamsulosin, folate, and vitamin D.**

**Pertinent physical exam included temperature of 100.1° Fahrenheit (F), heart rate 114 beats per minute, blood pressure 115/74 mmHg, and 100% oxygen saturation on room air. He had bilateral confluent purpura on the forearms and tenderness to palpation along the lumbar spine.**

Several other pertinent findings have been identified through the patient’s past history and examination. He has a history of tobacco use which is a risk factor for several malignancies. In addition, he is on several medications that could be contributing to his anemia. Diclofenac and other NSAIDs are associated with an elevated risk of gastrointestinal bleeding, and occult gastrointestinal bleed is still on our differential, particularly if subsequent laboratory investigations reveal microcytic anemia and iron deficiency.^5^ Oral methotrexate can cause bone marrow suppression particularly if the patient is having impaired clearance in the setting of a solitary kidney. Review of adverse event data from pertinent clinical trials of upadacitinib in psoriatic arthritis to gauge the likelihood of its contribution should be performed. The patient’s psoriatic arthritis could be associated with anemia of inflammation. The medications he is on (methotrexate, prednisone, upadacitinib) are immunosuppressive. That piece of information combined with tachycardia, temperature of 100.1 °F, and tenderness on examination of lumbar spine are also concerning for an infectious etiology such as discitis/osteomyelitis or bacteremia that has seeded the vertebrae. The point tenderness could also be indicative of a focal bony lesion from metastasis or primary malignancy. The purpura noted on exam makes me concerned for concurrent thrombocytopenia, disseminated infection, and/or coagulopathy. Along with the previously mentioned labwork, obtaining an LDH and haptoglobin to assess for laboratory evidence of hemolysis is needed. In addition, blood cultures along with imaging of the back should be considered from an infectious standpoint and a coagulation panel inclusive of PT/INR, PTT, and fibrinogen would help to assess for coagulopathy.

*The discussant is purposefully keeping a broad differential diagnosis at this point, while integrating the physical examination findings to identify the possibility for “don’t miss” diagnoses such as vertebral osteomyelitis or bacteremia.*

**Initial labs included a CBC with white blood cell (WBC) 3.1 K/µL with normal differential, Hgb 7.0 g/dL with MCV 94.1 fL, and platelets 147 K/µL. Metabolic panel was clinically unremarkable with normal kidney function (creatinine 1.02 mg/dL, BUN 18 mg/dL). Calcium was 8.1 mg/dL and phosphorus was 1.9 mg/dL. Hepatic panel was within normal limits. TSH was normal. Urinalysis was normal without evidence of microscopic hematuria. Blood cultures were pending. Chest X-ray was unremarkable.**

**Prior to admission, he was given one unit of packed red blood cells (PRBC) in the emergency department without appropriate response—repeat hemoglobin was 7.1 g/dL.**

The CBC demonstrates a normocytic anemia without the presence of other severe cytopenias. This is somewhat reassuring, although a primary bone marrow pathology cannot yet be ruled out. The degree of cytopenia can influence the probabilities of diagnoses on my differential. When there are very mild cytopenias (such as the thrombocytopenia and leukopenia noted here), sometimes they are driven by the acute issue prompting evaluation and quickly resolve once that issue is resolved. Common examples include a self-limited viral infection or sepsis. On the other hand, when the cytopenias are more severe (for example, an absolute neutrophil count persistently under 1000 or platelets under 100) then the likelihood of there being a primary etiology driving these cytopenias is higher. Given the degree of anemia is disproportionate to the drop in other cell lines, I would focus my attention there, while still being willing to return to a broader pancytopenia workup as incoming data dictates.

An inappropriate response to red cell transfusion is concerning for ongoing blood loss so this remains high on the differential. Brisk hemolysis could also contribute to an inappropriate response to transfusion; however, the bilirubin is normal, which is reassuring.

*One of the key skills that comes with clinical experience is being able to differentiate and apply nuance to specific findings. Rather than using a dichotomy of “pancytopenia” or not, the discussant is considering the “degree of cytopenia” to help “influence the probabilities of diagnoses” and determine if this is a primary pancytopenia problem. Interpreting studies using a probabilistic approach is critical to pursuing high-value care.* ^3^

**The patient was then given another unit of PRBCs with appropriate response to Hgb 8.3 g/dL. It was later learned on morning rounds that the first unit had mostly extravasated into the subcutaneous tissue due to infiltrated peripheral intravenous (IV) line, which was confirmed by physical examination demonstrating a large area of upper extremity edema and discoloration consistent with large-volume RBC extravasation.**

This update explains the initial inadequate response to RBC transfusion. I continue to be most concerned about a subacute/chronic source of blood loss or inadequate production of red blood cells.

*When a lab finding does not fit with the overall clinical picture, it is critical to further evaluate whether the result could be spurious, or if there was another issue leading to our conclusion. This highlights the importance of care coordination between clinical team-members—speaking directly with the bedside nurse and examining the patient avoided drawing false conclusions and pursuing unnecessary further work-up and management decisions.*

**Initial anemia labs now returned and were notable for absolute reticulocyte count of 0.024 10⁶ cells/µL (normal 0.025–0.09 10⁶ cells/µL), lactate dehydrogenase 337 U/L (normal 87–271 U/L), and haptoglobin 157 mg/dL (normal 43–212 mg/dL). Iron was 191 µg/dL, Ferritin 559 ng/mL, and Vitamin B12 838 pg/mL. Quantitative immunoglobulins and SPEP were normal. Parvovirus was negative. Blood cultures that were previously pending returned negative. The automated description of the peripheral blood smear included “metamyelocytes, myelocytes, smudge cells, giant thrombocytes, acanthocytes, and echinocytes.”**

**INR was 1.33 and Fibrinogen was 222 mg/dL (normal range: 253–390). D-dimer was significantly elevated at 14.98 µg/mL-FEU (normal range: 0–0.42). Given elevated D-dimer, tachycardia, and worsening dyspnea on exertion the week prior to admission, a CT angiography of the chest was obtained, which revealed pulmonary emboli with nonocclusive filling defects in the left and right main pulmonary arteries and all lobar and most segmental branches, without CT evidence of right heart strain** (Fig. 1)**.**

**Figure 1:**
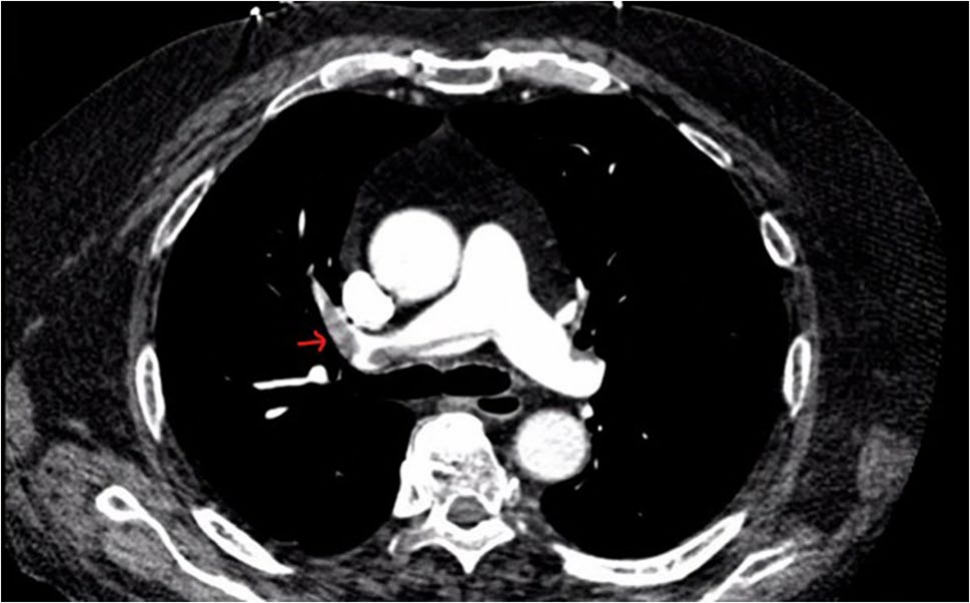
**CT angiogram of the chest with bilateral pulmonary emboli in the left and right main pulmonary arteries.**

**A CT of the lumbar spine demonstrated known multilevel chronic and/or degenerative changes, along with severe central canal narrowing at L3-L4, L4-L5, and moderate narrowing at L5-S1.**

A normal haptoglobin, mildly elevated LDH, normal bilirubin, along with a lack of schistocytes or spherocytes on peripheral smear, speak against hemolysis as the etiology of the anemia. LDH is non-specific for hemolysis, given its presence in several organ tissues, and elevations can happen with any etiology of cellular necrosis or turnover. The low reticulocyte response speaks to a hypoproliferative process; acute blood loss/hemolysis is unlikely to be the sole contributor to the patient’s anemia. Normal iron studies and B12 help to rule out nutritional etiologies. The immunoglobulin and SPEP being normal make a plasma cell dyscrasia less likely.

An automated report of a peripheral blood smear is inadequate and must be confirmed by pathologist review. However, the reported smear demonstrates several abnormal findings, including immature neutrophil precursors along with smudge cells. Immature neutrophil precursors can be seen in myeloid malignancies but can also be noted in patients that are acutely ill secondary to infection.^6^ The description of smudge cells is classically utilized to describe the lymphocytes seen in CLL or viral infections such as CMV; however, careful review of these cells should be done to confirm that they are not blasts or an artifact of slide preparation. Peripheral blood flow cytometry can help to better characterize these cells. The finding of giant platelets is nonspecific and can be seen in both myeloid malignancies and as a reactive finding. Echinocytes are described in patients with uremia but can also be a result of issues with slide preparation. In the context of our patient, the peripheral smear results support a hypoproliferative etiology and I am most concerned about a myeloid malignancy.

Lastly, our patient has an acute pulmonary embolism which raises my index of suspicion for underlying malignancy as a driver. I am most concerned for an underlying hematologic malignancy and getting a bone marrow biopsy will be necessary if an alternative etiology of anemia is not identified or the anemia does not improve.

*The discussant begins synthesizing the lab findings, noting that while some suggest one disease process, others point elsewhere, and not all results align clearly. A core high-value care principle is to avoid diagnostic cascades—chains of downstream testing and medical services triggered by an initial non-indicated test or an incidental finding. Thus, when results seem inconsistent or unexpected, clinicians should step back to evaluate whether further testing will meaningfully change management or simply add cost and potential harm. In addition, a diagnostic time-out is a high-value care tool that creates intentional space for clinicians to pause, reassess working diagnoses, and consider alternatives. For* *example, the temperature of 100.1°F led the discussant to consider infectious etiologies that, now with complete data, may be better explained by the later found pulmonary embolism.*

**After hematology-oncology consult review, the absolute number of atypical cells was determined to be highly unlikely to represent primary malignancy. Upon review of the patient’s psoriatic arthritis medications, methotrexate had been prescribed since 2001, but upadacitinib had been started 6 months prior to admission, which correlated with onset and gradual worsening of his pancytopenia.**

Drug-induced cytopenias can be difficult to definitively identify and oftentimes are a diagnosis of exclusion. Establishing a temporal relationship and ruling out alternative etiologies are all key steps in this process. A clinical trial of upadacitinib in psoriatic arthritis reported a 0.4–4.7% incidence of anemia dependent on the dose used,^7^ while longer follow-up from studies of its use in rheumatoid arthritis report an incidence of 15–20% of Grade 3 + anemia.^8^ Reticulocyte underproduction and the extent of abnormalities seen on the peripheral blood smear still make me quite concerned for a hypoproliferative etiology that is clonal. Clonal etiologies would include the spectrum of aggressive hematologic malignancies (i.e., acute leukemia, large cell lymphoma), more indolent malignancies (i.e., MDS or myelofibrosis), and pre-malignant conditions (i.e., clonal cytopenias of undetermined significance (CCUS)). The hypoproliferative findings on the peripheral smear would also be consistent with marrow suppression from a drug-induced anemia, though I would recommend getting a bone marrow biopsy with the appropriate ancillary testing (cytogenetics, molecular testing) to rule out an underlying clonal disorder before attributing the anemia exclusively to upadacitinib. This can be pursued as an outpatient assuming there is clinical improvement during admission, particularly since many of the ancillary studies from a bone marrow biopsy can take several days to result.

The practice of high-value care hinges on evidence-based decision-making, using the available evidence and likelihoods to help guide next steps. The discussant is drawing on the available literature to determine the likelihood that upadacitinib could explain this level of anemia. He is balancing the potential benefits and risks of pursuing further testing.

*The practice of high-value care hinges on evidence-based decision-making, using the available evidence and likelihoods to help guide next steps. The discussant is drawing on the available literature to determine the likelihood that upadacitinib could explain this level of anemia. He is balancing the potential benefits and risks of pursuing further testing.*

**Upadacitinib and methotrexate were both stopped in the hospital due to suspicion for drug-induced anemia. Following conversation with the patient about risks and benefits, bone marrow biopsy was deferred in the inpatient setting in favor of monitoring his response to cessation of these medications. He was also started on apixaban for pulmonary embolism. Two weeks after discharge, complete blood count showed improvement in all cell lines: WBC 10 K/µL, Hgb 9.3 g/dL, and Plt 318 K/µL.Furthermore, peripheral blood smear showed resolution of previously reported myelocytes and metamyelocytes. His CBC remained stable (Hgb back at his prior chronic baseline of above 10 g/dL) at hematology outpatient follow-up 5 months after discharge and was attributed to anemia of chronic inflammation from psoriatic arthritis (ferritin 559 ng/mL). Given that his hemoglobin returned to baseline, his outpatient hematologist chose to defer bone marrow biopsy and monitor blood counts every 2 months.**

The improvement in hemoglobin back to baseline is quite reassuring. While the patient’s hemoglobin is not back to normal, there may be a component of chronic inflammation from his psoriatic arthritis. The anemia was thought to be drug-related, and there was improvement with holding the potential offending medications. While the patient’s clinical course does not support an aggressive hematologic malignancy, such as large cell lymphoma or acute leukemia, more indolent clonal disorders such as CCUS and MDS remain on the differential, particularly given an incidence of up to 10% of clonal hematopoiesis in adults aged 70–79.^9,10^

The only way to definitively assess for these etiologies is performing a bone marrow biopsy with appropriate cytogenetic and molecular testing. Given the patient’s improvement, however, it is appropriate to defer the biopsy to the outpatient setting with close monitoring, assuming that a nuanced conversation has occurred with the patient regarding the alternative etiologies that can only be ruled in/out with a bone marrow biopsy. In particular, lower-risk MDS and CCUS can be monitored without therapeutic information and the most valuable component of making the diagnosis comes from prognostic data that can inform overall survival and the risk of disease progression.^11,12^

*The discussant recognizes the diagnostic uncertainty in this case and highlights the importance of continuing to follow and consider further diagnostic testing to explain remaining abnormalities. Diagnostic uncertainty is a key challenge in clinical practice, arising from nonspecific or overlapping symptoms, limitations of diagnostic tests and their characteristics, evolving clinical presentations, and incomplete information.*^13^ *Even when a presumptive “discharge diagnosis” has been made, it remains important to not fully anchor on this diagnosis and to have contingency planning about potential next steps. Key to these decisions is transparent communication with patients and setting expectations. Deciding on whether a test such as a bone marrow biopsy should be performed in the hospital versus outpatient is a common tension in high-value care practice.*^14^

## DISCUSSION

This case illustrates how high-value care principles can guide diagnostic reasoning in the setting of significant uncertainty. The patient’s acute-on-chronic anemia, mild accompanying cytopenias, and atypical peripheral smear findings raised concern for serious underlying pathology, including hematologic malignancy. However, careful attention to probabilities, temporal relationships, and response to initial management helped avoid premature diagnostic closure.

The temporal association between worsening anemia and upadacitinib initiation led to a presumptive diagnosis of medication-induced pancytopenia. Upadacitinib can suppress bone marrow via inhibition of the JAK/STAT pathway that is involved in normal hematopoiesis.^15^ In our patient with chronic anemia at baseline, the degree of anemia observed was disproportionally greater compared to other cell lines. The JAK/STAT pathway is also involved in the expression of various critical mediators of cancer and inflammation.^15^ While the FDA lists venous thromboembolism (VTE) as a black box warning for some JAK inhibitors, recent meta-analyses do not show a clear increased risk.^16,17^ As JAK inhibitors become more common as targeted immunomodulatory therapies across a range of inflammatory and hematologic conditions, clinicians must be alert to adverse effects like infection, thrombosis, and myelosuppression.

The case also highlights the importance of diagnostic time-outs when data appear discordant. The initially inadequate response to transfusion could have prompted additional testing or escalation of care; instead, reassessment revealed a technical explanation that resolved the discrepancy and prevented a diagnostic cascade. Similarly, automated peripheral smear findings were interpreted cautiously, with expert consultation and absolute cell counts guiding next steps rather than descriptive terminology alone.

High-value care does not mean withholding necessary testing, but rather aligning testing with patient-specific risk, anticipated benefit, and downstream consequences. In this case, deferring inpatient bone marrow biopsy in favor of close outpatient follow-up reflected an intentional balance between diagnostic certainty and potential harm.

Ultimately, this case underscores that diagnostic uncertainty is an inherent feature of clinical medicine. High-value care provides a framework for managing that uncertainty through thoughtful test selection, ongoing reassessment, clear communication, and avoidance of low-yield interventions.

### CLINICAL TEACHING POINTS

- JAK inhibitors are increasingly used for various inflammatory and hematologic conditions, so clinicians must recognize risks such as myelosuppression and potential VTE.
- Atypical smear findings should be interpreted with the clinical context and absolute counts
- If medication-induced myelosuppression is suspected, bone marrow biopsy may be deferred in favor of close monitoring.
- High-value care in diagnostic uncertainty involves using structured frameworks, stepwise workups, thoughtful test selection, and judicious use of time.

## Data Availability

N/A
